# A method for identifying moonlighting proteins based on linear discriminant analysis and bagging-SVM

**DOI:** 10.3389/fgene.2022.963349

**Published:** 2022-08-15

**Authors:** Yu Chen, Sai Li, Jifeng Guo

**Affiliations:** College of Information and Computer Engineering, Northeast Forestry University, Harbin, China

**Keywords:** moonlighting proteins, protein recognition, machine learning, linear discriminant analysis, bagging-SVM

## Abstract

Moonlighting proteins have at least two independent functions and are widely found in animals, plants and microorganisms. Moonlighting proteins play important roles in signal transduction, cell growth and movement, tumor inhibition, DNA synthesis and repair, and metabolism of biological macromolecules. Moonlighting proteins are difficult to find through biological experiments, so many researchers identify moonlighting proteins through bioinformatics methods, but their accuracies are relatively low. Therefore, we propose a new method. In this study, we select SVMProt-188D as the feature input, and apply a model combining linear discriminant analysis and basic classifiers in machine learning to study moonlighting proteins, and perform bagging ensemble on the best-performing support vector machine. They are identified accurately and efficiently. The model achieves an accuracy of 93.26% and an F-sorce of 0.946 on the MPFit dataset, which is better than the existing MEL-MP model. Meanwhile, it also achieves good results on the other two moonlighting protein datasets.

## 1 Introduction

With the continuous expansion of proteomic data and the continuous study of protein functions by researchers, multifunctional proteins have gradually attracted people’s attention. Among multifunctional proteins, people have found a new type of protein that can perform multiple functions autonomously without partitioning these into separate domains, and they are called moonlighting proteins (MPs) (Huberts et al., 2010). Under the influence of certain specific factors, such as cell localization, cell type, substrate or different cofactor, moonlighting proteins can switch their executive functions (Jeffery, 1999). At present, moonlighting proteins have been found in a variety of animals, plants and microorganisms, and a large number of studies have shown that moonlighting proteins play an important role in organisms. They can be used as enzymes for catalytic reactions, as well as secreted cytokines, transcription factors and DNA stabilizers. Through the study of moonlighting proteins, it is found that they can play an important role in the development of new therapies for some diseases (Jeffery, 2018). For example, moonlighting proteins can be used as targets for active medicines in the treatment of hepatitis B virus, cancer, and bacterial infections (Adamo et al., 2021; Zakrzewicz and Geyer, 2022). Due to the excellent performance of moonlighting proteins in disease treatment, the discovery of new moonlighting proteins is of great significance for solving many medical problems. Therefore, the prediction of moonlighting proteins has become a hot research direction.

At present, there are several online available moonlighting protein databases that can obtain protein sequences. Jeffery’s laboratory manually collected some strict moonlighting proteins from peer journals, and built a searchable and Internet-based MoonProt database, which has been updated to MoonProt 3.0 (Chen C. et al., 2021). Luis et al. constructed a multi-functional protein database MultitaskProtDB, designed to provide a free online database for researchers using bioinformatics methods to predict multifunctional proteins, and has been updated to MultitaskProtDB-II (Franco-Serrano et al., 2018). Bo et al. established PlantMP, the first plant moonlighting protein database, enabling researchers to conveniently collect and process plant-specific raw data (Su et al., 2019).

Based on these public moonlighting protein databases, researchers have constructed several models to predict moonlighting proteins. In 2016, Khan and Kihara et al. developed a moonlighting protein prediction model called MPFit, which achieved 98% accuracy when protein gene ontology (GO) annotations were available, and 75% accuracy using omics features when no GO annotations were available (Khan and Kihara, 2016). In 2017, Khan et al. proposed a new solution: they built DextMP based on three types of textual information of proteins (title, abstracts from literature and function description in UniProt) and machine learning classifier, achieving 91% accuracy (Khan et al., 2017). In 2021, Li et al. proposed a multimodal deep ensemble learning architecture called MEL-MP. Firstly, they extracted four sequence-based features: primary protein sequence information, evolutionary information, physical and chemical properties, and secondary protein structure information; secondly, they selected a specific classifier for each feature; finally, they applied stacked ensemble to integrate the output of each classifier. The method showed excellent predictive performance, which achieved an F-score of 0.891 (Li et al., 2021). In the same year, Shirafkan et al. constructed a new moonlighting protein dataset to identify MPs and non-MPs through the SVM method of SAAC feature, and established a well-judged scheme to detect outlier proteins (Shirafkan et al., 2021). Liu et al. believed that an appropriate method was needed to identify plant moonlighting proteins, so they used the combination of Tri-Peptide composition (TPC) and XGBoost to construct IdentPMP, which was a plant moonlighting protein prediction tool (Liu et al., 2021).

For MPFit and DextMP, although high accuracy can be obtained, GO annotations and text information of protein samples need to be provided, which is very restrictive. Other experiments have shortcomings such as relatively low model accuracy and low efficiency due to the complexity of the model (Li et al., 2021; Shirafkan et al., 2021). In order to solve the above problems, we propose a new scheme. Firstly, we extract the SVMProt-188D feature, which contains information of protein composition and eight physicochemical properties that are effective in showing the properties of moonlighting proteins (Zou et al., 2016). Secondly, linear discriminant analysis (LDA) is used to reduce the dimensionality of the feature set to achieve separation of positive and negative samples. Finally, bagging ens is performed on SVM to classify moonlighting proteins. The main contributions of this paper are as follows: 1) We propose a method combining LDA and Bagging-SVM to classify moonlighting proteins. 2) We conduct extensive experiments on MPFit dataset, Shirafkan’s dataset, and plant moonlighting protein dataset, and the model achieves excellent performance on these datasets.

## 2 Materials and methods

**FIGURE 1:**
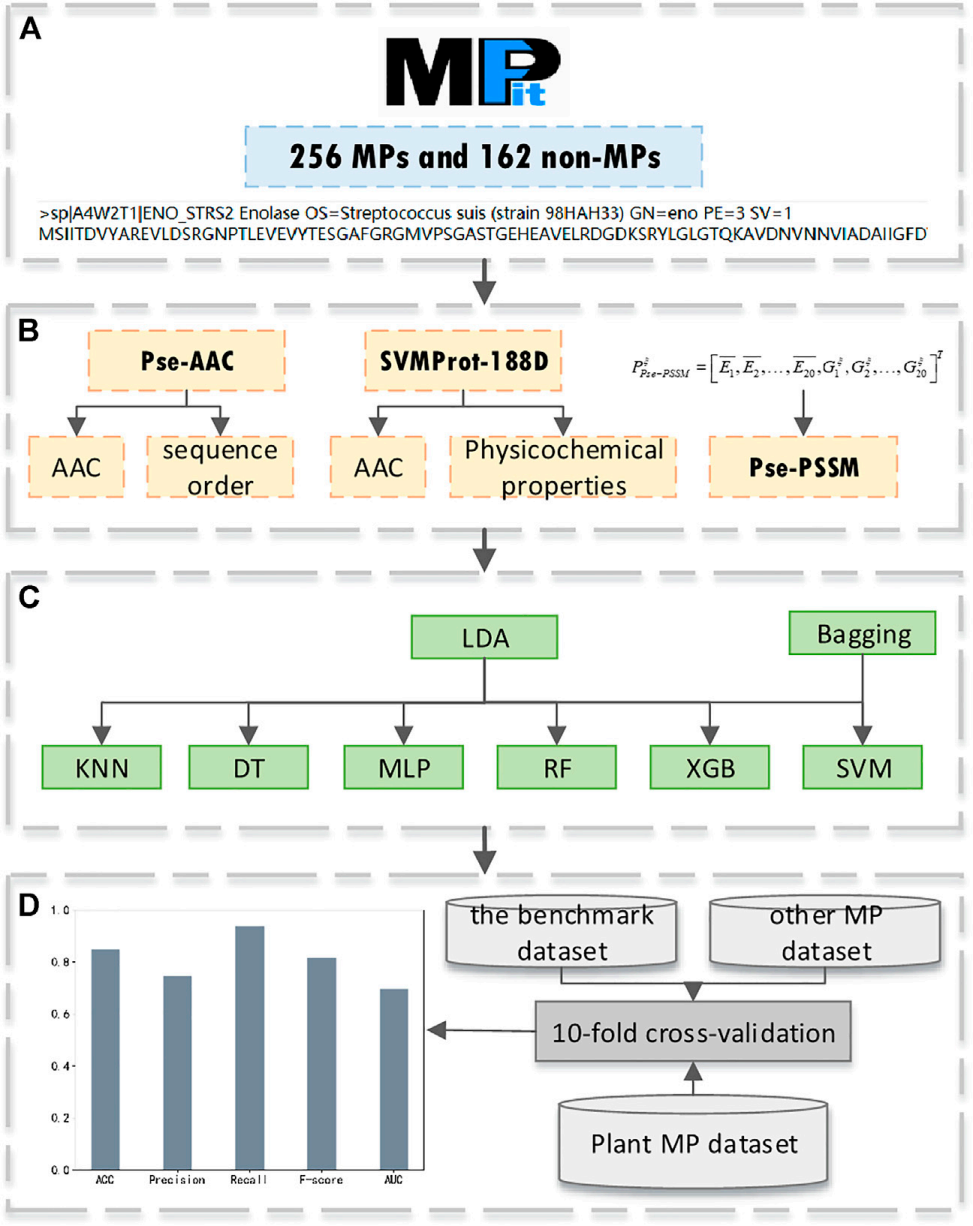
The pipeline of our experiment, **(A)** benchmark dataset acquisition; **(B)** feature extraction; **(C)** model construction; **(D)** model evaluation.

Our research is mainly divided into four parts: benchmark dataset acquisition; feature extraction; model construction; model evaluation. The experimental process is shown in Figure 1. Firstly, we use MPFit as the benchmark dataset (a). Secondly, we extract SVMProt-188D as a feature and compare the classification results of this feature with Pse-AAC and Pse-PSSM (b). Thirdly, we combine LDA with Bagging-SVM for protein classification, and compare the classification results with other base classifiers to verify the superiority of the classifier (c). Finally, we use multiple datasets to validate our method and compare the classification results with state-of-the-art models to demonstrate the effectiveness of our method (d).

### 2.1 Benchmark dataset

In this study, we use the benchmark dataset constructed by Khan and Kihara et al. (MPFit dataset) [9]. The dataset contains 268 MPs and 162 non-MPs. The positive examples in the dataset are derived from 268 proteins with Uniprot ID extracted from MoonProt database, and their biological origins are shown in Table 1(Mani et al., 2015). Screening of suitable proteins from four genomes of human, E. coli, yeast and mouse as negative example of moonlighting proteins (single-function proteins). The screening criterias are as follows: 1) target protein with at least eight GO term annotations; 2) when clustering GO terms in the biological process (BD) category using a semantic similarity score threshold between 0.1 and 0.5, no more than one cluster is obtained at each threshold; 3) there is no more than one cluster of GO terms for molecular function (MF) with semantic similarity scores between 0.1 and 0.5. After removing non-MPs with more than 25% similarity to MPs, 162 negative samples were obtained (Table 1) (Khan and Kihara, 2016). This dataset has been used in several experiments on moonlighting protein prediction and is very authoritative in the field (Khan and Kihara, 2016; Khan et al., 2017; Li et al., 2021; Shirafkan et al., 2021). Therefore, it is suitable as the benchmark dataset for this study. Also, we have conducted experiments on the state-of-the-art dataset of Shirafkan et al. (2021).

**TABLE 1:** Composition of the benchmark dataset.

Organism	MPs		Non-MPs	
	Number	Percentage (%)	Number	Percentage (%)
Human	45	16.8	60	37.0
*Escherichia* *coli*	30	11.19	16	9.88
Yeast	27	10.1	34	20.9
Mouse	11	4.1	52	32.1
Other	155	57.81	0	0.0
Total	268	100	162	100

### 2.2 Feature extraction

Feature extraction is a crucial step in the process of identifying proteins. This process is the conversion of the amino acid sequence of a protein into discrete data of a certain length, and the representation of a sample of the protein by features composed of discrete data. At present, a variety of features have been used in the study of protein classification, such as amino acid composition, positional information, physicochemical properties, evolutionary information and secondary structure. Pse-AAC, SVMProt-188D and Pse-PSSM reflect positional information, physicochemical properties and evolutionary information respectively, which is important for protein recognition. Therefore, we choose these three features as the feature vectors of this study. The details are as follows.

#### 2.2.1 Pse-AAC

Since the amino acid composition does not take into account the influence of sequence order information, the researchers propose the feature of pseudo-amino acids (Pse-AAC). The feature combines regular amino acid composition (frequency of occurrence of 20 amino acids) with a set of discrete sequence correlation factors, which are primarily used to address the problem that sequence information cannot be directly incorporated into the prediction algorithm due to different lengths of amino acid sequences (Chou, 2001; Ding et al., 2009; Tang et al., 2016; Awais et al., 2021). The specific descriptions are as follows.

$$X={\left[x_{1}\cdots x_{20},x_{20+1}\cdots x_{20+\lambda }\right]}^{T}$$

Where *X* represents Pse-AAC, $x_{1}$ to $x_{20}$ represent the regular amino acid composition, and $x_{20+1}$ to $x_{20+\lambda }$ represent the information of sequence order. $x_{i}$ in *X* is expressed as follows.

$$x_{i}=\{\begin{array}{c} \frac{f_{i}}{\sum_{j=1}^{20}f_{j}+\omega \sum_{k=1}^{\lambda }\theta_{k}}\left(1\leq i\leq 20\right)\\ \frac{\omega \theta_{i-20}}{\sum_{j=1}^{20}f_{j}+\omega \sum_{k=1}^{\lambda }\theta_{k}}\left(20+1\leq i\leq 20+\lambda \right) \end{array}$$

Where $f_{i}$ is the frequency of occurrence of the 20 amino acids, $\theta_{k}$ is the k-layer sequence correlation factor, and ω is the weighting factor for sequence order effects, ω = 0.05 in our study. The λ components can be defined by the user at will (Yan et al., 2020). In this experiment, hydrophilic, hydrophobic, mass, pK1, pK2, pI, rigidity, flexibility, and irreplaceability are added, resulting in a 65-dimensional feature vector.

#### 2.2.2 SVMProt-188D

The SVMProt-188D includes the frequency of 20 amino acids (i.e., “ACDEFGHIKLMNPQRSTVWY”) and eight physical and chemical properties (hydrophobicity, normalized van der Waals volume, polarity, polarizability, charge, secondary structure, solvent accessibility, and surface tension) (Cai et al., 2003). The details are shown in Table 2, and will be introduced separately below.

**TABLE 2:** Eight physical and chemical properties of the 188-dimensions.

Attribute	Division		
hydrophobicity	Polar:RKEDQN	Neutral:GASTPHY	Hydrophobicity:CVLIMFW
Normalized van der waals volume	Small:GASCTPD	Medium:NVEQIL	Large:MHKFRYW
polarity	Low:LIFWCMVY	Medium:PATGS	High:HQRKNED
polarizability	Low:GASDT	Medium:GPNVEQIL	High:KMHFRYW
charge	Positive:KR	Neutral:ANCQGHILMFPSTWYV	Negative:DE
Secondary structure	Helix:EALMQKRH	Strand:VIYCWFT	Coil:GNPSD
Solvent accessibility	Buried:ALFCGIVW	Exposed:RKQEND	Intermediate:MPSTHY
Surface tension	Large:GQDNAHR	Medium:KTSEC	Small:ILMFPWYV

The frequency of 20 amino acids can be calculated by the following formula:

$$F_{i}=\frac{N_{i}}{L},\;\;\left(i=A,C,D,\ldots ,Y\right)$$

Where $N_{i}$ is the number of amino acid type *i*, and *L* is the length of a protein sequence.

Eight physicochemical properties are studied on the composition, transition, and distribution of amino acids, and each property is divided into three groups (Dubchak et al., 1995; Wang et al., 2017; Xiong et al., 2018; Zou et al., 2019).

##### 2.2.2.1 Composition

Taking the hydrophobicity attribute as an example, “RKEDQN” is polar, “GASTPHY” is neutral, and “CVLIMFW” is hydrophobic. The frequency of each group can be expressed as:

$$C_{i}=\frac{N_{i}}{L},\;\;i\in \left\{polar,neutral,hydrophobic\right\}$$

##### 2.2.2.2 Transition

The transition from polar group to neutral group is the frequency of polar residue following neutral residue or neutral residue following polar residue. The transition between neutral group and hydrophobic group, and the transition between hydrophobic group and polar group have similar definitions. It can be expressed by the following formula:

$$T\left(i_{1},i_{2}\right)=\frac{N\left(i_{1},i_{2}\right)+N\left(i_{2},i_{1}\right)}{L-1},\;\;\left(i_{1},i_{2}\right)\in \left\{\left(polar,neutral\right),\left(neutral,hydrophobic\right),\left(hydrophobic,polar\right)\right\}$$

##### 2.2.2.3 Distribution

The distribution represents the position of the first, 25%, 50%, 75%, and last of each group category in the amino acid sequence.

#### 2.2.3 Pse-PSSM

Inspired by Pse-AAC signatures, and combining with evolutionary information, Chou et al. proposed a new signature, Pse-PSSM (Chou and Shen, 2007; Wang et al., 2020). The original PSSM profile $P_{PSSM}$ was generated by running the position-specific iterative basic local alignment search tool (PSI-BLAST) against Uniref50 database, and setting the E-value to 0.001 for 3 iterations (Ding et al., 2014).

$$P_{PSSM}=\left[\begin{array}{ccc} E_{1\to 1} & \cdots & E_{1\to 20}\\ \vdots & \ddots & \vdots \\ E_{L\to 1} & \cdots & E_{L\to 20} \end{array}\right]$$

Where $E_{i\to j}$ represents the score of the amino acid residue at the i-th position of the protein sequence being changed to amino acid residue type *j* during the evolutionary process, *L* is the length of the protein sequence, *k* from 1 to 20 indicate the 20 natural amino acid types. Implement the following standardised procedures:

$$E_{i\to j}=\frac{E_{i\to j}^{0}-\frac{1}{20}\sum_{k=1}^{20}E_{i\to k}^{0}}{\sqrt{\frac{1}{20}\sum_{u=1}^{20}{\left(E_{i\to j}^{0}-\frac{1}{20}\sum_{k=1}^{20}E_{i\to k}^{0}\right)}^{2}}}$$

In order to make the dimension size of the PSSM descriptors consistent, the following operations are performed:

$$\bar{P_{PSSM}}={\left[\bar{E_{1}},\bar{E_{2}},\cdots ,\bar{E_{20}}\right]}^{T}$$

$$\bar{E_{j}}=\frac{1}{L}\overset{L}{\underset{i=1}{\sum }}E_{i\to j}$$

Where $\bar{E_{j}}$ is the average score of the i-th amino acid in the protein sequence *P* over the course of biological evolution. In order to preserve sequence order information, the concept of pseudo-amino acid composition is used to obtain the final 40-dimensional Pse-PSSM by considering the correlation between two amino acids $E_{i\to j}$ .

$$P_{Pse-PSSM}^{\xi }={\left[\bar{E_{1}},\bar{E_{2}},\cdots ,\bar{E_{20}},G_{1}^{\xi },G_{2}^{\xi },\cdots ,G_{20}^{\xi }\right]}^{T}$$

$$G_{j}^{\xi }=\frac{1}{L-\xi }\overset{L-\xi }{\underset{i=1}{\sum }}{\left[E_{i\to j}-E_{\left(i+\xi \right)\to j}\right]}^{2}$$

### 2.3 Feature selection

**FIGURE 2:**
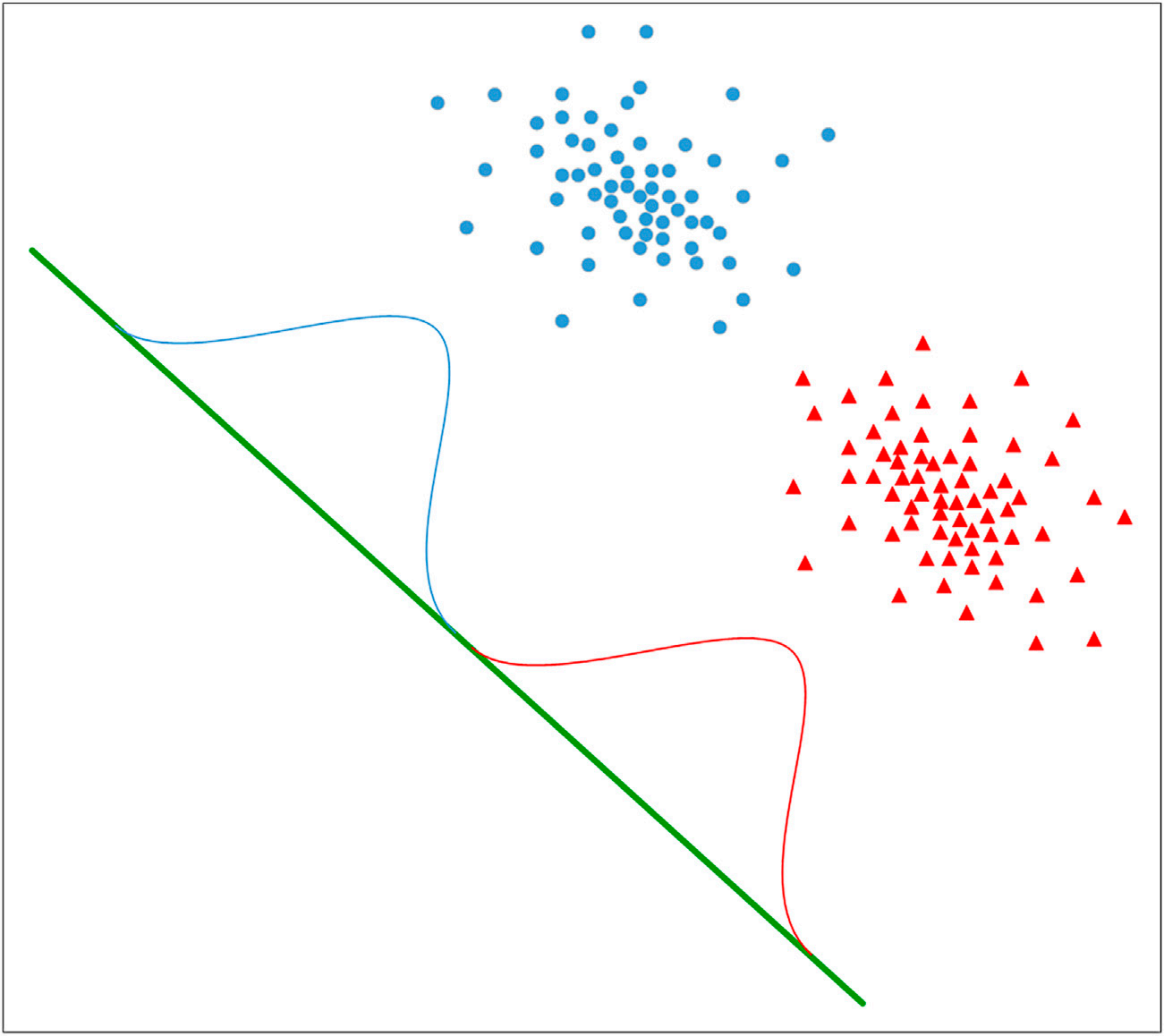
The diagram of LDA applied to a binary classification algorithm.

Linear discriminant analysis (LDA) is a feature selection technique (Arjmandi and Pooyan, 2012; Xie et al., 2018; Yang et al., 2020; Chen Y. et al., 2021). It can effectively reduce the feature dimension and reduce the error caused by redundant data. The idea of LDA is to project samples from high-dimensional space onto low-dimensional space where the distance between samples of the same category is minimized and the distance between samples of different categories is maximized, thus making the samples more easily distinguishable and obtaining better classification results. Therefore, this study uses LDA for dimensionality reduction. The diagram of LDA applied to a binary classification algorithm is shown in Figure 2.

#### 2.3.1 The linear discriminant analysis process is as follows

Suppose we have *N* protein samples which can be denoted as $\left\{\left(x_{1},y_{1}\right),\;\left(x_{2},y_{2}\right),\cdots ,\left(x_{n},y_{n}\right)\right\}$ , where $x_{i}$ is the features of the protein sample and $y_{i}$ is the label of the protein sample, $y_{i}\in \left(0,1\right)$ . Our aim is to find a projection line *W* such that the projection $Y=W^{T}x_{i}$ of sample $x_{i}$ on the line minimizes the intra-class distance and maximizes the inter-class distance. Firstly, calculate the mean vector for each class:

$$\mu_{j}=\frac{1}{N_{j}}\underset{x\in X_{j}}{\sum }x\left(j=0,1\right)$$

Where $N_{j}$ is the number of samples of class *j* and $X_{j}$ is the set of samples of class *j*, $\mu_{j}$ is the mean vector of the j-th class of samples.

Then, calculate the within-class scatter matrix $S_{W}$ :

$$S_{W}=\underset{0}{\sum }+\underset{1}{\sum }=\underset{x\in X_{0}}{\sum }\left(x-\mu_{0}\right){\left(x-\mu_{0}\right)}^{T}+\underset{x\in X_{1}}{\sum }\left(x-\mu_{1}\right){\left(x-\mu_{1}\right)}^{T}$$

Where $\underset{j}{\sum }$ is the covariance matrix of samples of class *j* (strict lack of covariance matrix of the numerator), expressed by the following formula:

$$\underset{j}{\sum }=\underset{x\in X_{j}}{\sum }\left(x-\mu_{j}\right){\left(x-\mu_{j}\right)}^{T}\left(j=0,1\right)$$

Calculating the between-class scatter matrix $S_{B}$ :

$$S_{B}=\left(\mu_{0}-\mu_{1}\right){\left(\mu_{0}-\mu_{1}\right)}^{T}$$

Finally, the optimization objective is:

$$arg\;max\;J\left(W\right)=\frac{W^{T}S_{B}W}{W^{T}S_{W}W}$$

Simplify the above formula to get the target projection line $W^{*}$ :

$$W^{*}=arg\;max\left\{\frac{W^{T}S_{B}W}{W^{T}S_{W}W}\right\}=S_{W}^{-1}\left(\mu_{0}-\mu_{1}\right)$$

The original set of samples is projected onto the one-dimensional space W to obtain the 1-dimensional feature vector after dimensionality reduction (Chen Y. et al., 2021).

### 2.4 Classifier

In the experiments, we use six popular base classifiers, including K-nearest-neighbor (KNN) (Deng et al., 2016), Decision Tree (DT) (Safavian and Landgrebe, 1991), Multilayer Perceptrons (MLP) (Lee et al., 2020), Random Forests (RF) (Breiman, 2001), XGBoost (Chen et al., 2016; Chen et al., 2020) and Support Vector Machine (SVM). Experimental parameters for all classifiers can be found in Supplementary Table S1. After evaluation on the benchmark dataset, the support vector machine works best, and can avoid overfitting when the number of samples is small (Gong et al., 2021). Through bagging ensemble of SVM, the model performance is further improved.

SVM is a type of supervised learning proposed by Vladimir Vapnik and is widely used in machine learning, computer vision and data mining, such as image recognition, text classification and protein sequence classification (Zhao et al., 2015; Ding et al., 2017; Manavalan et al., 2018; Zhang et al., 2019). In binary classification problems, the main idea of SVM is to find a segmentation hyperplane that maximizes the distance of the segmentation hyperplane from the nearest point. Given a training sample $x_{i}\in R^{P}$ , i = 1, … , n, and a vector $y\in {\left\{0,1\right\}}^{n}$ , our goal is to find $w\in R^{P}$ and b∈R for a given prediction $sign\left(w^{T}\phi \left(x\right)+b\right)$ that predicts correctly for most samples. In this experiment, we use the SVC algorithm for classification and set the kernel function to linear function and the penalty parameter *C* to 1.0.

Bagging is a common ensemble learning method that integrates the prediction results of multiple base classifiers into the final strong classifier prediction result. Its integration strategy is to obtain training subsets by sampling from the original sample set, and each training subset trains a model. Finally, the classification results of samples are obtained by voting strategy (Breiman, 1996; Zaman and Hirose, 2008).

### 2.5 Performance assessment

We used these indicators to evaluate the performance of the experiment: accuracy (ACC), Precision, Recall, F-score and AUC (area of ROC curve) (Wei et al., 2017; Shan et al., 2019; Basith et al., 2020; Zhang et al., 2020; Wang et al., 2021). These evaluation indicators are the results of the confusion matrix calculation obtained from the experiment, and the calculation formulas are as follows:

$$ACC=\frac{TP+TN}{TP+TN+FP+FN}$$

$$Precision=\frac{TP}{TP+FP}$$

$$Recall=\frac{TP}{TP+FN}$$

$$F-score=\frac{2}{\frac{1}{Precision}+\frac{1}{Recall}}=\frac{2*Precision*Recall}{Precision+Recall}$$

Where TP represents the number of correctly predicted MPs, TN represents the number of correctly predicted non-MPs, FP represents the number of incorrectly predicted MPs as non-MPs, and FN represents the number of incorrectly predicted non-MPs as MPs.

## 3 Results and discussion

### 3.1 Performance evaluation of different feature extraction

**FIGURE 3:**
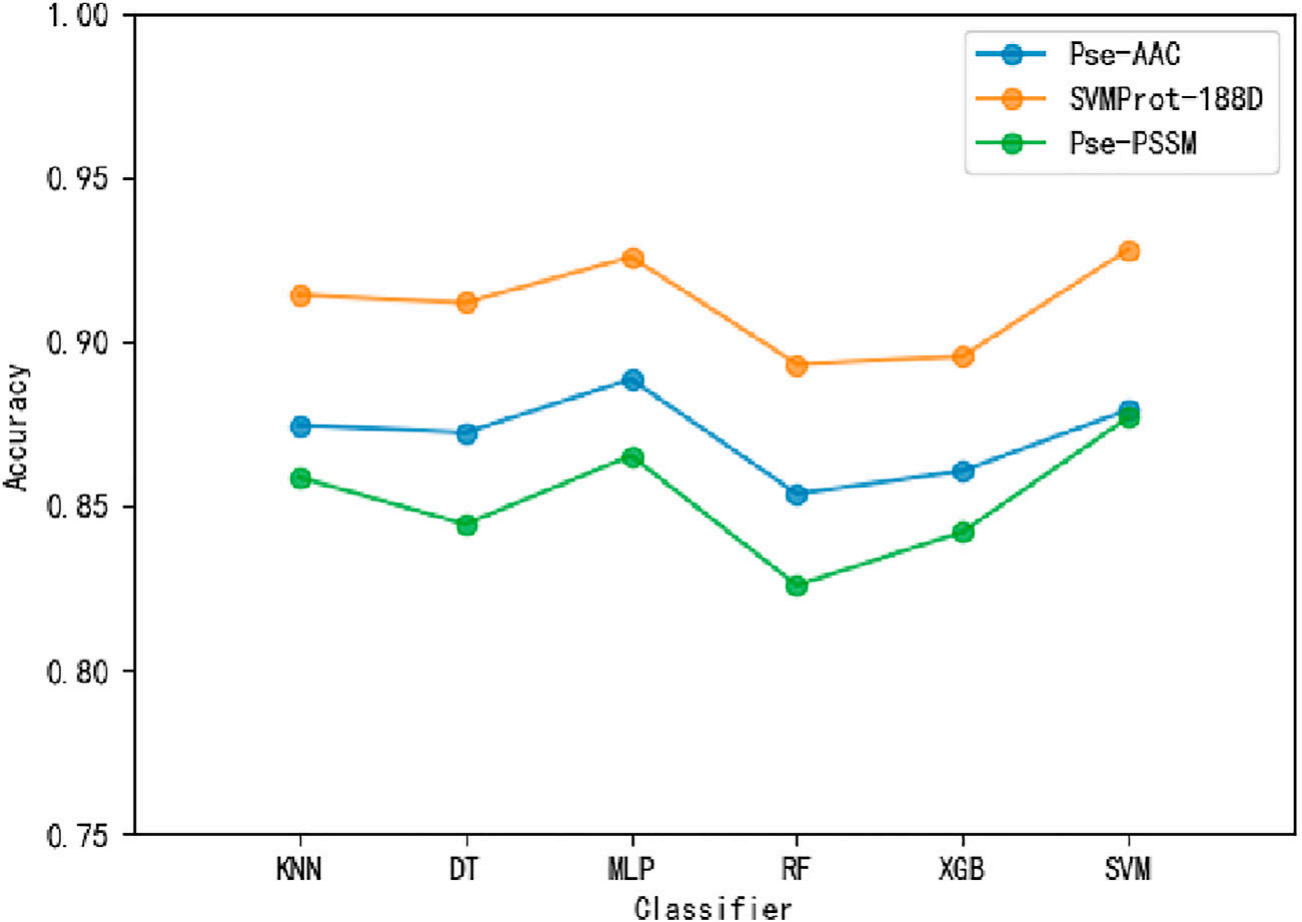
The accuracy of different features in each classifier.

To ensure the accuracy of the experimental results, the 10-fold cross-validation (i.e., The training samples are divided into ten folds, nine of which are adopted for training, one of which is adopted for testing. The process repeats 10 times and the average value is taken as the final result.) is applied on the benchmark dataset. To select suitable input data, Pse-AAC, SVMProt-188D and Pse-PSSM are experimented with multiple classifiers respectively (Table 3). It is clear from the table that the SVMProt-188D performs best on all indicators, with the most accuracy rates exceeding 90% (Figure 3). In contrast, Pse-AAC and Pse-PSSM don’t perform as well as SVMProt-188D. From this, we hypothesize that: On the one hand, MPs can change their functions under certain conditions, such as substrate concentration or cofactor change, and there are great differences in physicochemical properties between them and non-MPs; on the other hand, SVMProt-188D is a linear feature of the protein, which can be easily identified by the classifier after LDA.

**TABLE 3:** The results of 10-fold cross-validation using a variety of classifiers and hybrid features.

Feature	Method	ACC (%)	Precision	Recall	F-score	AUC
Pse-AAC	KNN	87.4419	0.885	0.923	0.901	0.863
	DT	87.2093	0.892	0.909	0.898	0.865
	MLP	88.8372	0.898	0.931	0.912	0.878
	RF	85.3488	0.883	0.887	0.883	0.847
	XGB	86.0465	0.891	0.891	0.888	0.856
	SVM	87.907	0.9	0.913	0.904	0.872
SVMProt-188D	KNN	91.3953	0.919	0.944	0.931	0.906
	DT	91.1628	0.918	0.946	0.929	0.906
	MLP	92.5581	0.939	0.941	0.939	0.922
	RF	89.3023	0.917	0.911	0.912	0.891
	XGB	89.5349	0.92	0.911	0.914	0.893
	SVM	92.7907	0.943	0.942	0.942	0.925
Pse-PSSM	KNN	85.8514	0.886	0.886	0.884	0.848
	DT	84.4189	0.884	0.868	0.872	0.839
	MLP	86.5116	0.917	0.868	0.888	0.869
	RF	82.5581	0.858	0.862	0.858	0.815
	XGB	84.186	0.869	0.883	0.873	0.833
	SVM	87.6744	0.921	0.883	0.898	0.879

### 3.2 Performance evaluation of different classifiers

**FIGURE 4:**
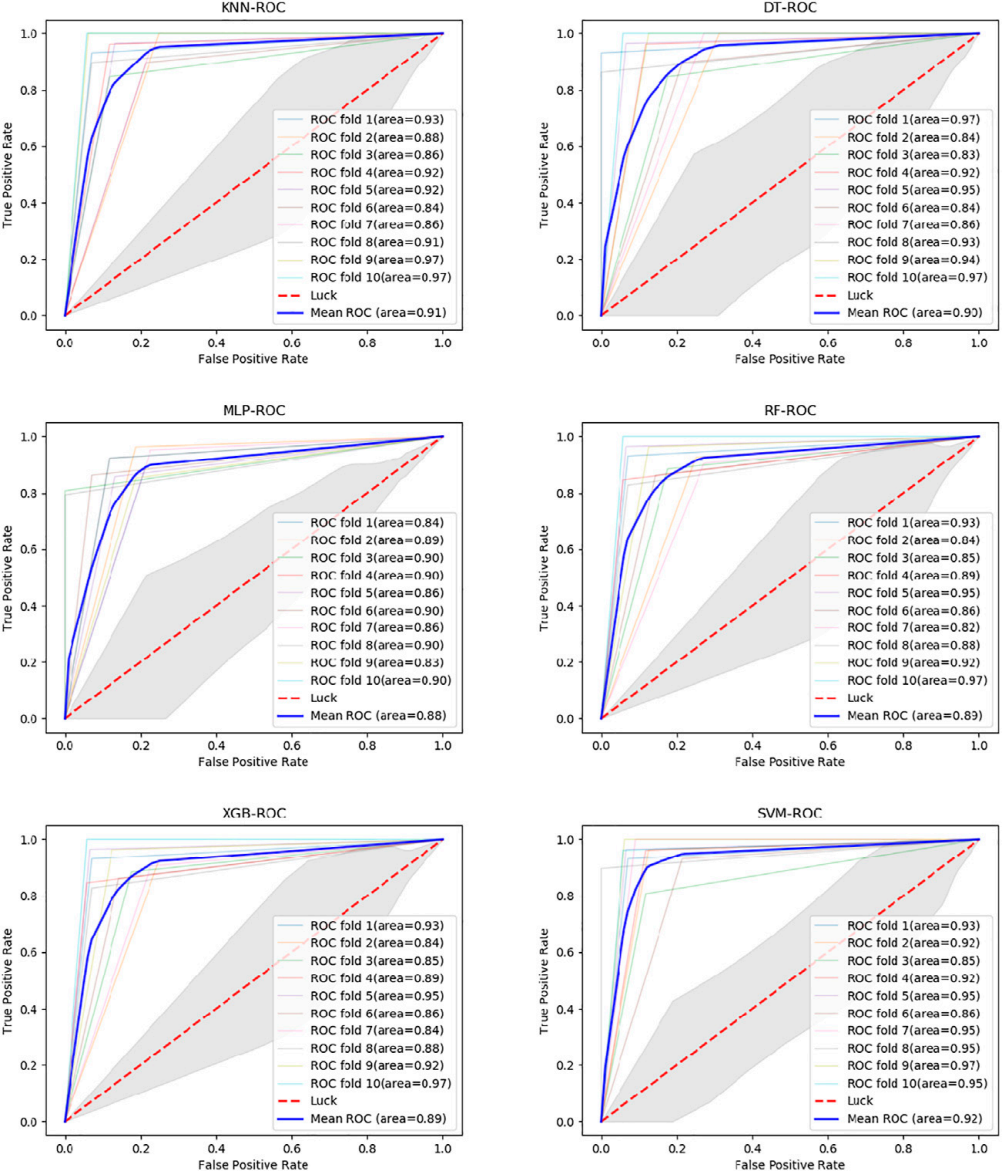
ROC curves of different classifiers on SVMProt-188D.

**FIGURE 5:**
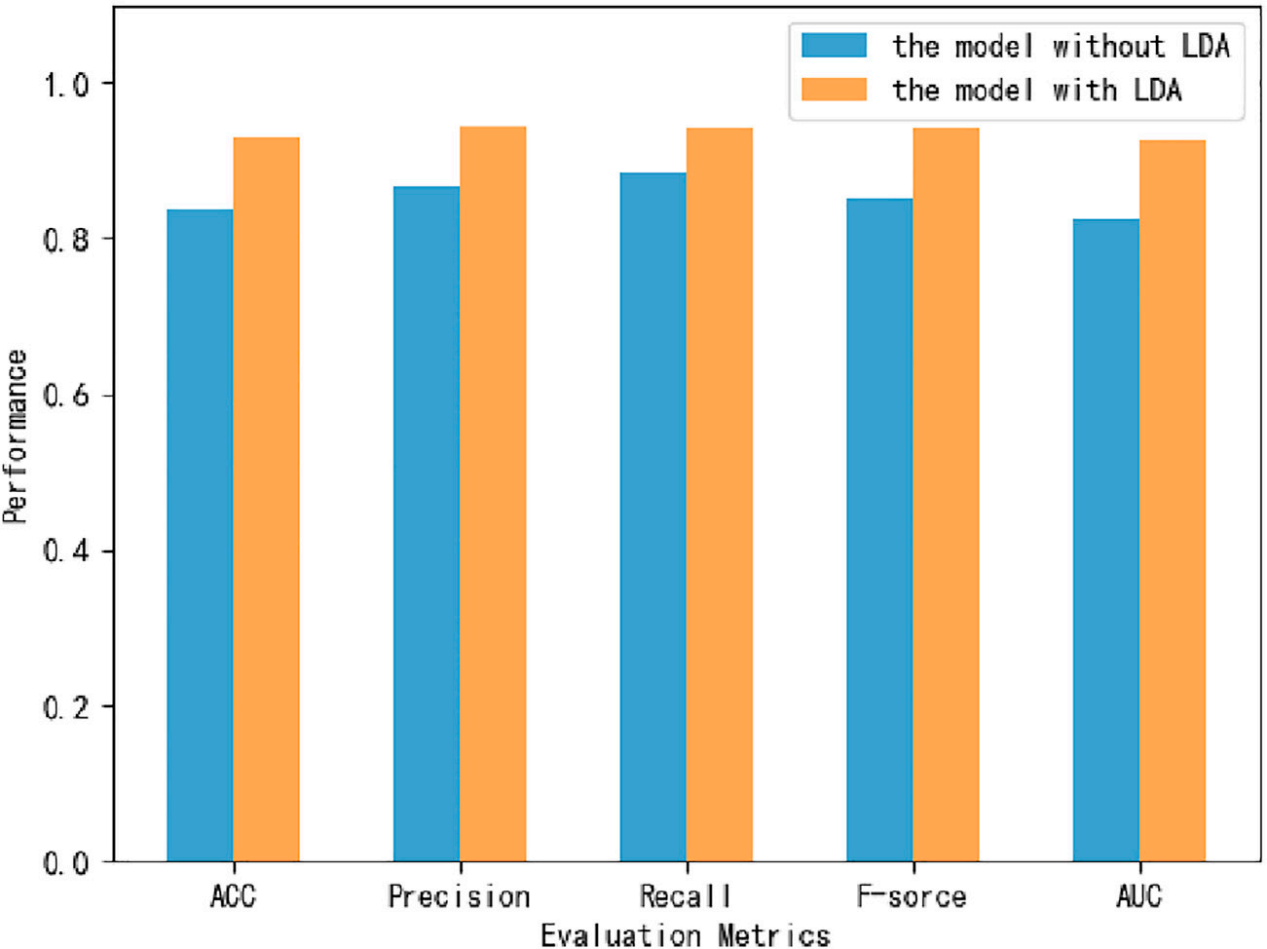
The performance of the model after and before the implementation of LDA.

Six classifiers from scikit-learn are used in this study for comparison experiments, namely KNN, DT, MLP, RF, XGBoost, and SVM. From the data, SVM obtains an accuracy rate of 92.7907%, which is the highest accuracy rate. Despite the unbalanced benchmark dataset used in this experiment, with 268 positive and 162 negative samples, the classifier achieves high scores of 0.943, 0.942 and 0.925 on the three metrics of percision, F-score and AUC (Figure 4). The MLP is second only to the SVM and also achieves high scores in various metrics. Of these, surprisingly, DT obtains the highest recall value, 0.946. Because we use accuracy as the main metric, SVM is the most suitable classifier for this experiment. Furthermore, we compare this model with the model without LDA (Figure 5). From the figure, we can observe that the LDA dimensionality reduction method has greatly improved the experimental results, proving that it is very effective in the identification of MPs.

### 3.3 Comparison of Bagging-SVM and single SVM

The above experiments prove that the combination of SVMProt-188D and support vector machine has the best effect. Based on the excellent performance of bagging ensemble algorithm in the field of machine learning, we use bagging to integrate SVM and verify the classification performance of the integrated model (Chen and Association for Computing Machinery, 2018; Kaur et al., 2019; Raihan-Al-Masud and Mondal, 2020). The results are shown in Table 4 (The experimental results of bagging integration with all classifiers can be obtained from the Supplementary Figures S1, S2). As can be seen from the table, ACC, Precision, Recall, F-score and AUC all improved, which indicates that Bagging-SVM is effective for the classification of moonlighting proteins. Bagging-SVM can reduce the error caused by a single support vector machine to the experimental results, improving the stability of the model, and have stronger convincing.

**TABLE 4:** The results of Bagging-SVM and Single SVM.

Method	ACC (%)	Precision	Recall	F-score	AUC
SVM	92.7907	0.943	0.942	0.942	0.925
Bagging_SVM	93.2558	0.944	0.949	0.946	0.928

### 3.4 Comparison with other methods

We compare with the more current MP classification models, including Khan’s MPFit (Khan and Kihara, 2016), Li’s MEL-MP(Li et al., 2021) and Shirafkan’s method (Shirafkan et al., 2021). The results of the comparison are shown in Table 5 (Where '*' is for data not given in the comparison papers). The experimental results for all three models above are obtained with the MPFit dataset, mostly using 10-fold cross-validation. Therefore, they are very suitable for comparison with our model. As can be observed from the table, our model outperforms the other prediction methods on all the remaining evaluation indicators except for the AUC. In particular, the F-score of 0.946 is 5.4% higher than the second highest, MEL-MP (F-score = 0.892).

**TABLE 5:** Comparison with other methods.

Method	ACC (%)	Precision	Recall	F-score	AUC
MPFit	75	*	*	0.784	*
MEL-MP	*	0.895	0.893	0.892	0.947
Shirafkan’s	81.7	0.813	*	0.802	0.806
Our	92.7907	0.943	0.942	0.942	0.925

### 3.5 Performance on other MPs datasets

To verify that our model can effectively classify moonlighting proteins, we obtain a state-of-the-art moonlighting protein dataset from Shirafkan’s paper, which includes 215 positive samples and 136 negative samples (Shirafkan et al., 2021). Similarly, feature extraction is performed on this dataset to obtain SVMProt-188D features, and then, using 10-fold cross-validation, classification is performed on our model. In order to verify the generalization ability of our model, MPFit dataset is used as the training set and Shirafkan’s dataset is used as the independent testing set to conduct the experiment again. The experimental results are shown in Table 6. Method 1 is the result of 10-fold cross-validation, and method 2 is the result of independent testing. On this dataset, we still obtain an accuracy rate higher than 91%, and the other four indicators also achieve high scores, proving that our model has a strong generalization ability.

**TABLE 6:** The results of other dataset on our model.

Method	ACC (%)	Precision	Recall	F-score	AUC
Method1	91.1746	0.91	0.949	0.929	0.901
Method2	91.4530	0.907	0.958	0.932	0.902

**FIGURE 6:**
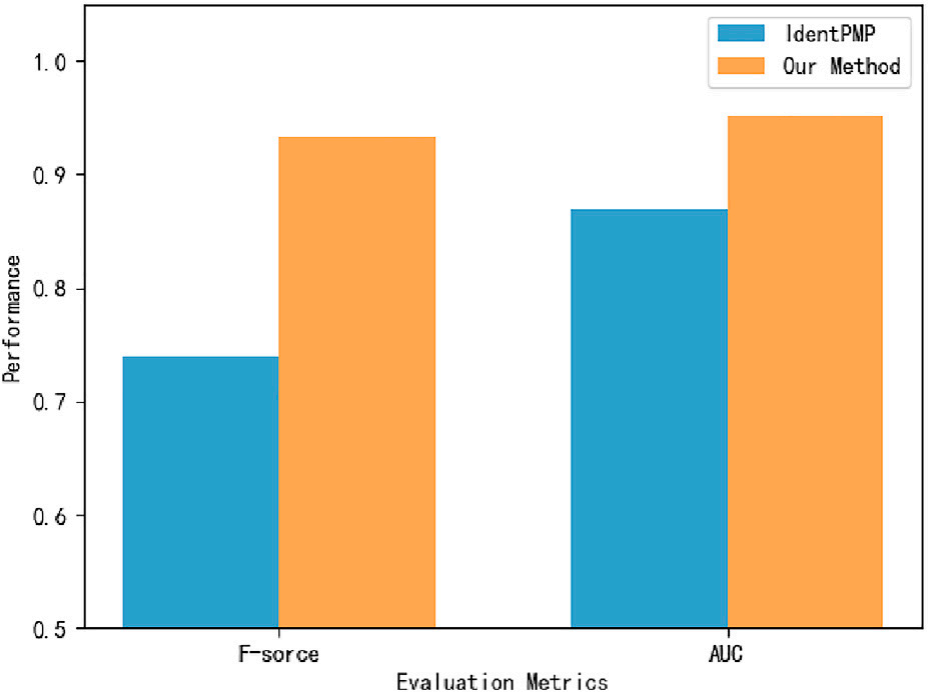
The performance of the plant MPs dataset on our model.

To verify that the model can effectively classify plant moonlighting proteins, we obtain the Uniprot ID of the plant moonlighting protein dataset from Liu et al. and obtain protein sequences from the corresponding databases according to the UniprotID (Liu et al., 2021). In order to compare with IdentPMP, 10-fold cross-validation is used on the same dataset, and the experimental results are shown in Figure 6. On the dataset of plant MP, the accuracy of 94.9692% is obtained by 10-fold cross-validation, far exceeding IdentPMP in F-score and AUC.

## 4 Conclusion

In this paper, we propose a method for identifying moonlighting proteins based on bagging-SVM ensemble learning classifier. Firstly, feature extraction is carried out on the collected benchmark dataset, and after comparison, SVMprot-188D is selected. Then, we use the feature selection method of LDA to reduce the dimension of the feature. Finally, the Bagging-SVM ensemble learning algorithm is used to construct the prediction model. The experimental results show that our model achieves good results in various indicators and is superior to the current advanced models. In order to prove that our model has strong generalization ability, we also use the dataset in Shirafkan’s paper to conduct experiments, and the accuracy rate has exceeded 91%. In addition, plant MPs are found to be equally applicable to our method, which is a great improvement compared with the previous experimental method. However, the depth of machine learning model is relatively shallow. In the future, we will try to use deep learning model to identify MPs, and hope to make new breakthroughs in this field.

## Data Availability

The datasets presented in this study can be found in online repositories. The names of the repository/repositories and accession number(s) can be found in the article/Supplementary Material.
