# Supplementary material for: A method for identifying moonlighting proteins based on linear discriminant analysis and bagging-SVM
Source: Front Genet. 2022 Aug 15;13:963349. doi: 10.3389/fgene.2022.963349 (PMC9420859; doi:10.3389/fgene.2022.963349)
Supplement: Supplementary file 1 [file DataSheet2.pdf]

Table S1 Parameters for each classifier.

classifier	parameter
KNN	K=3
DT	max_depth=5
MLP	solver='lbfgs',hidden_layer_sizes=(50,50),max_iter=100, learning_rate_init=0.1, alpha=1e-4
RF	n_estimators=250
XGB	max_depth=7,min_child_weight=5,learning_rate=0.05, n_estimators=100
SVM	C=1.0, kernel='linear'
