# Supplementary figures and images for: A method for identifying moonlighting proteins based on linear discriminant analysis and bagging-SVM
Source: Front Genet. 2022 Aug 15;13:963349. doi: 10.3389/fgene.2022.963349 (PMC9420859; doi:10.3389/fgene.2022.963349)

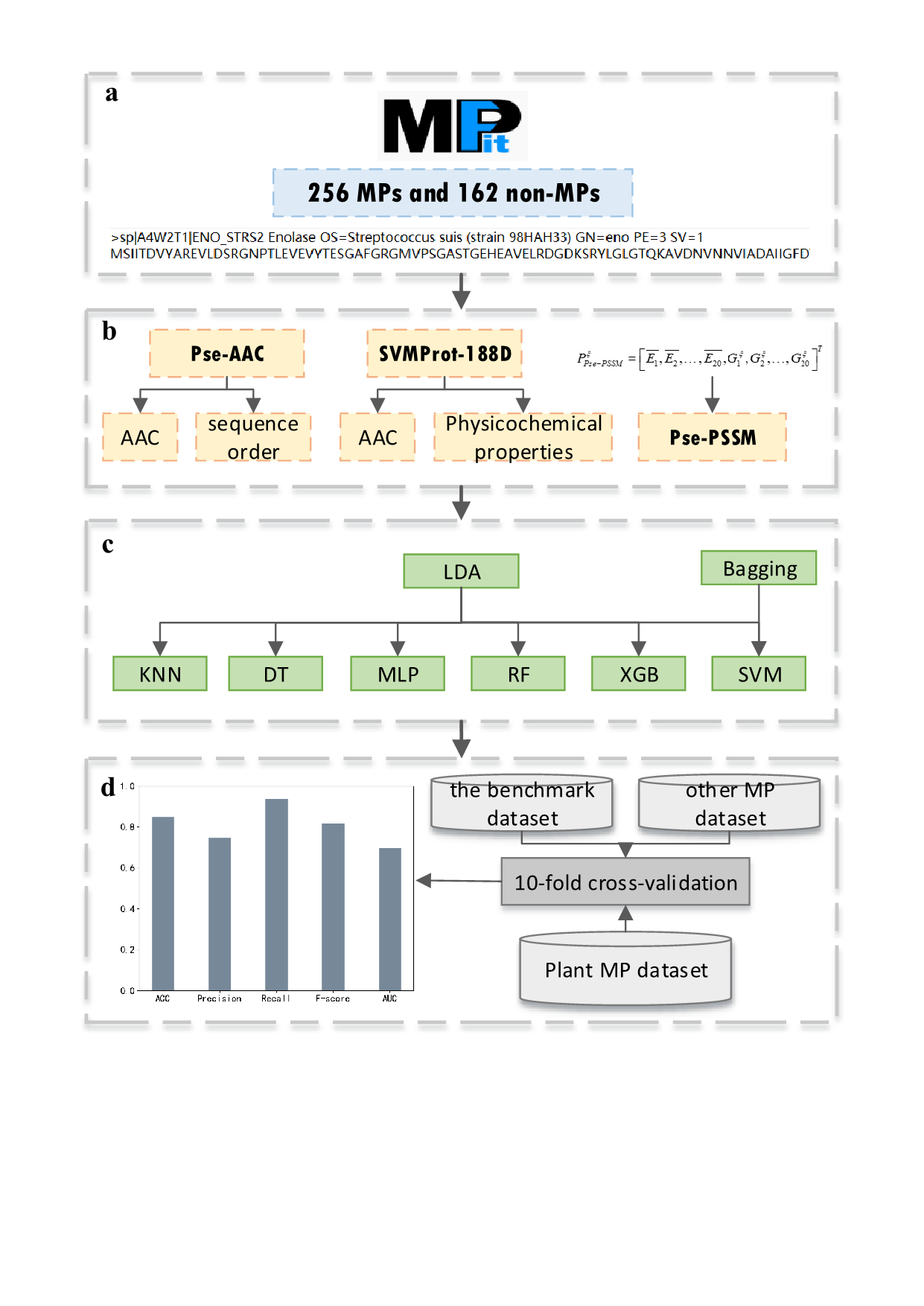

Supplement: Supplementary file 3 [file DataSheet4.zip › Figures/Figure1.tif]

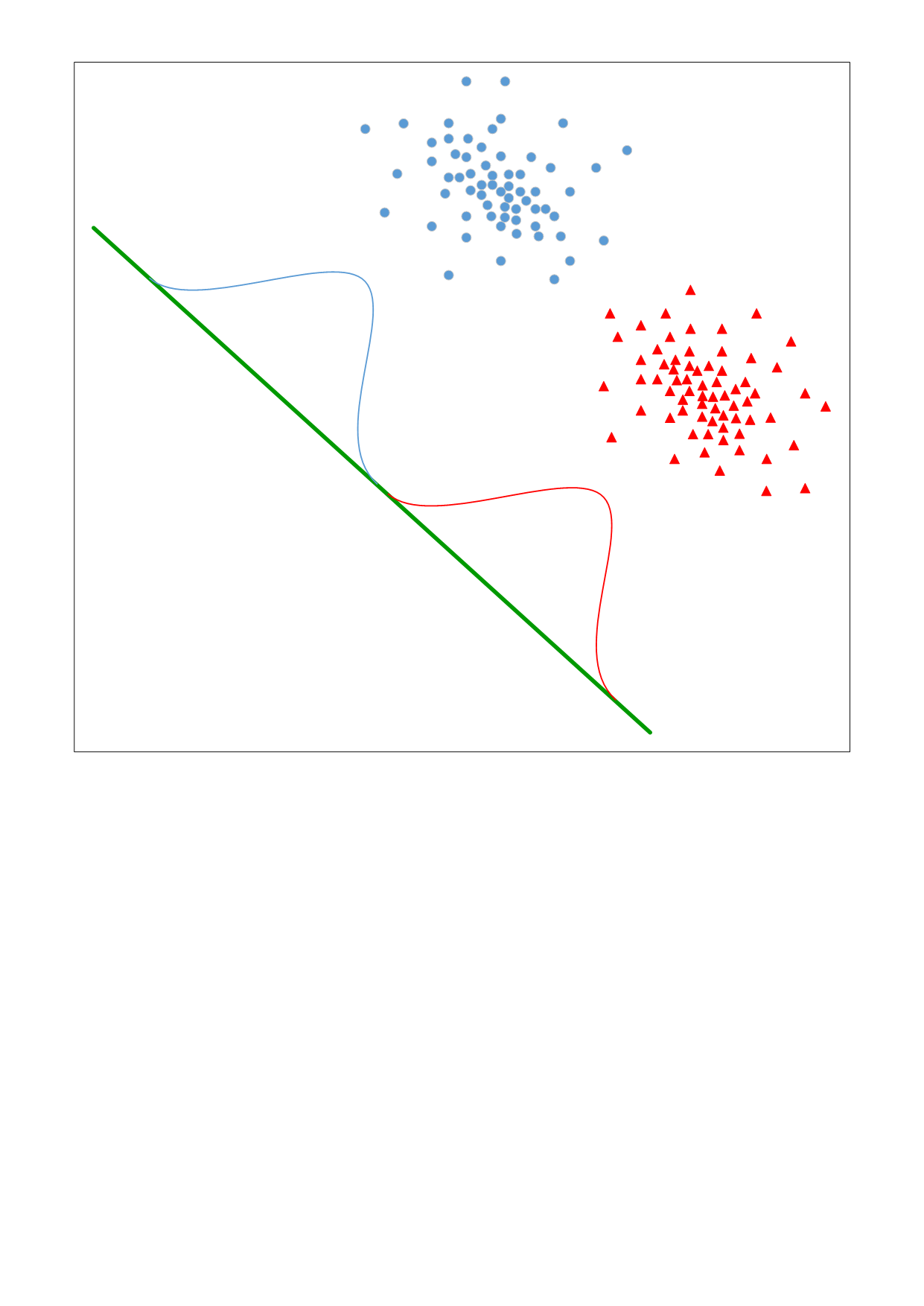

Supplement: Supplementary file 3 [file DataSheet4.zip › Figures/Figure2.tif]

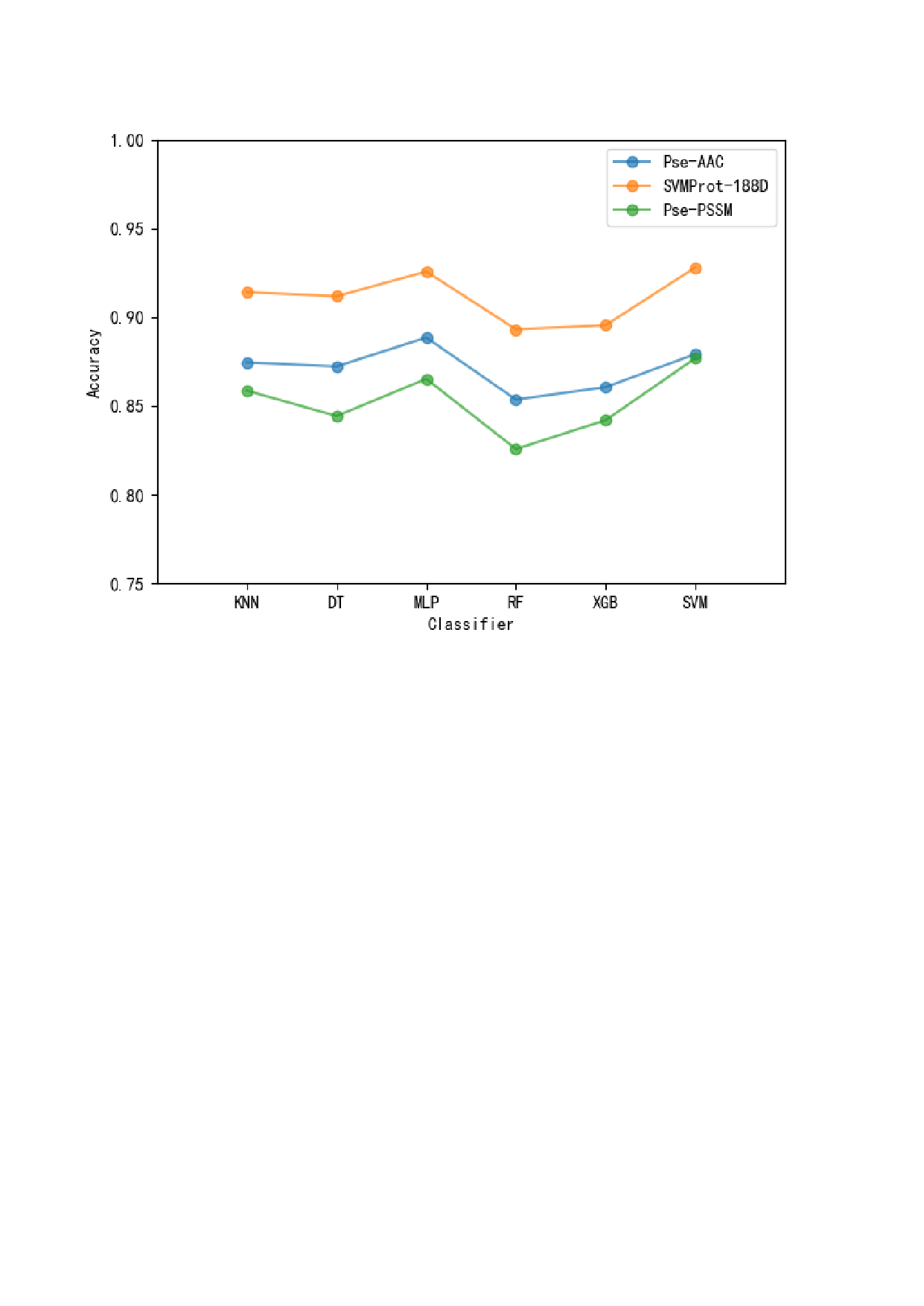

Supplement: Supplementary file 3 [file DataSheet4.zip › Figures/Figure3.tif]

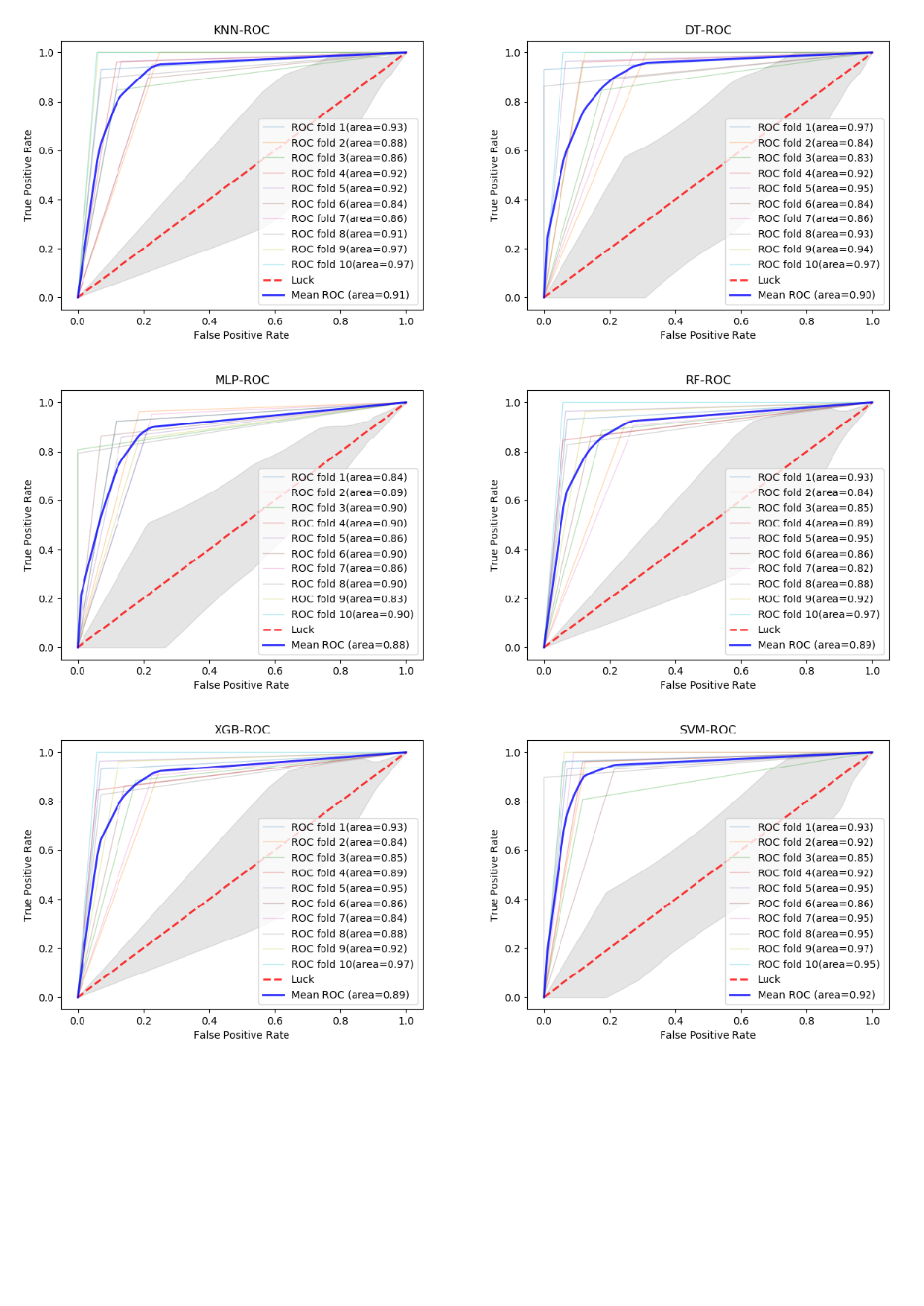

Supplement: Supplementary file 3 [file DataSheet4.zip › Figures/Figure4.tif]

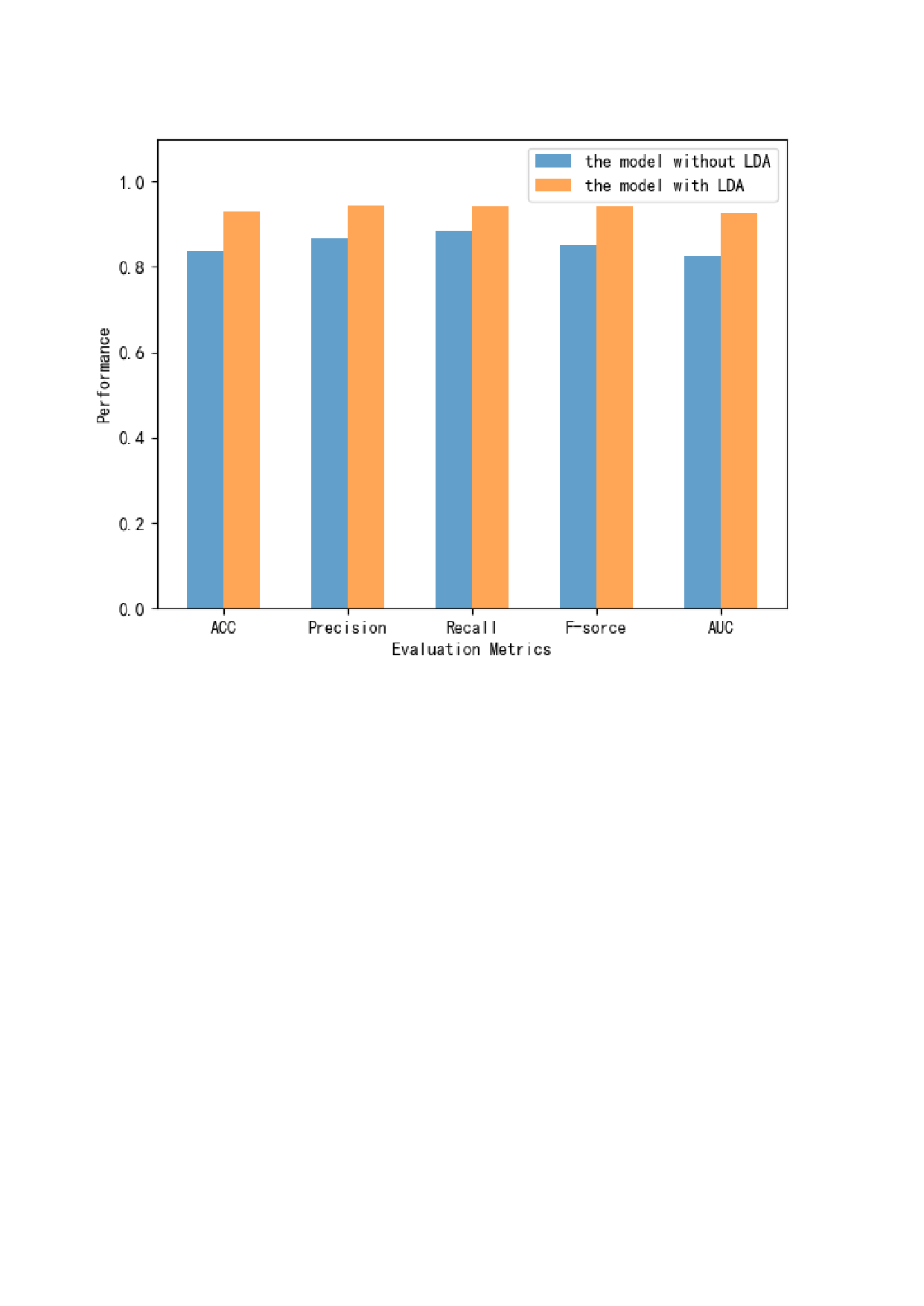

Supplement: Supplementary file 3 [file DataSheet4.zip › Figures/Figure5.tif]

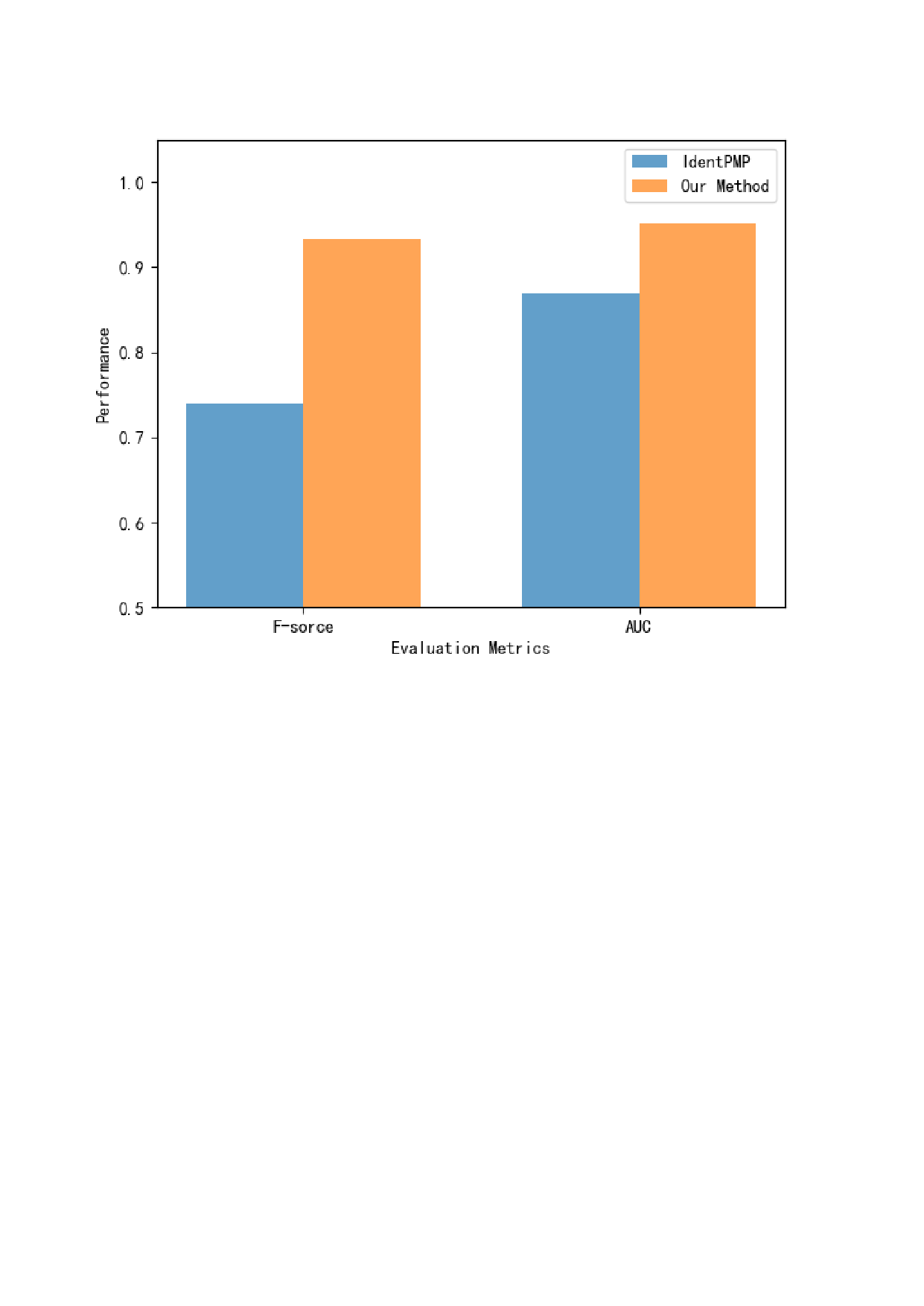

Supplement: Supplementary file 3 [file DataSheet4.zip › Figures/Figure6.tif]

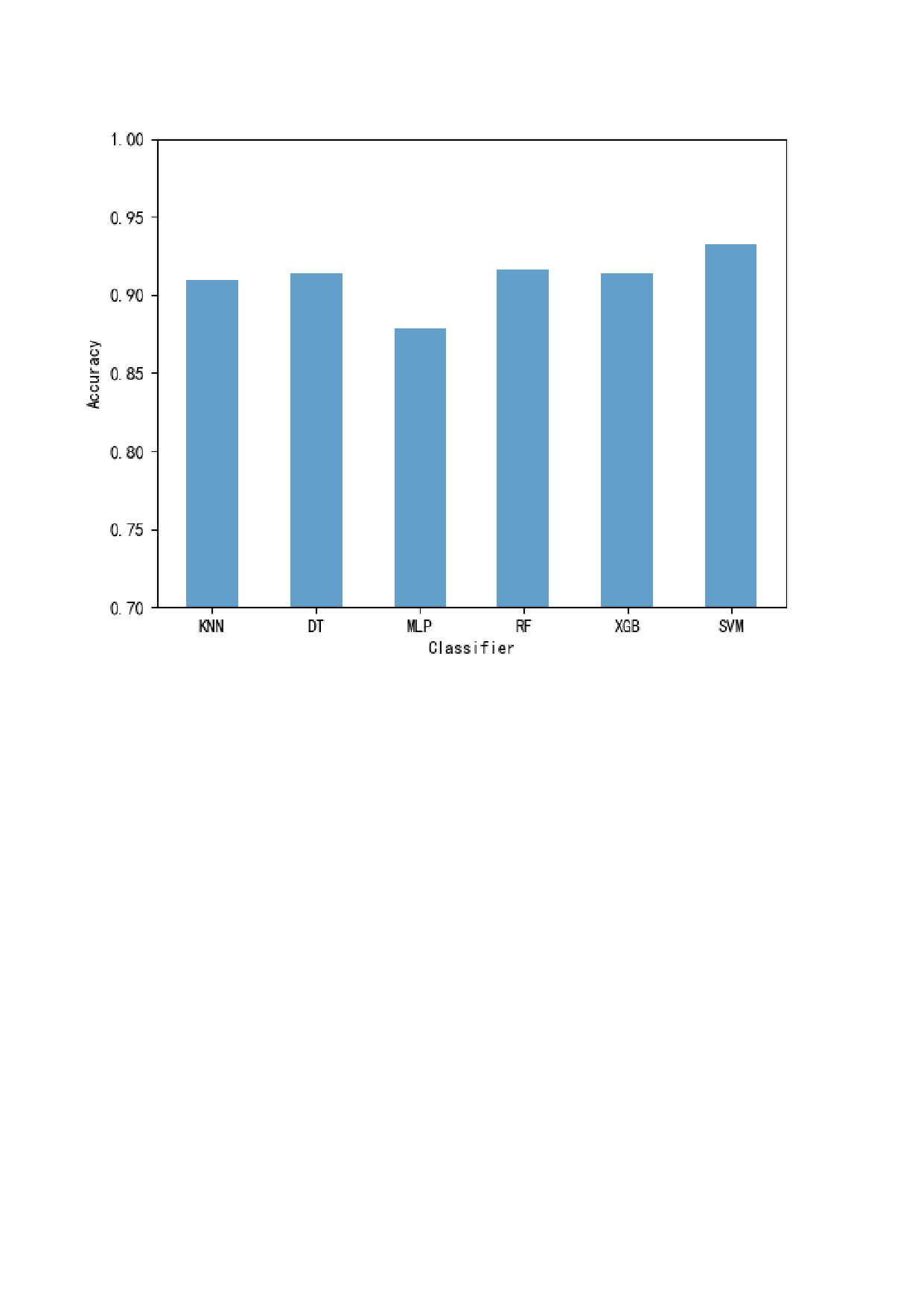

Supplement: Supplementary file 3 [file DataSheet4.zip › Figures/Supplementary Figure S1.tif]

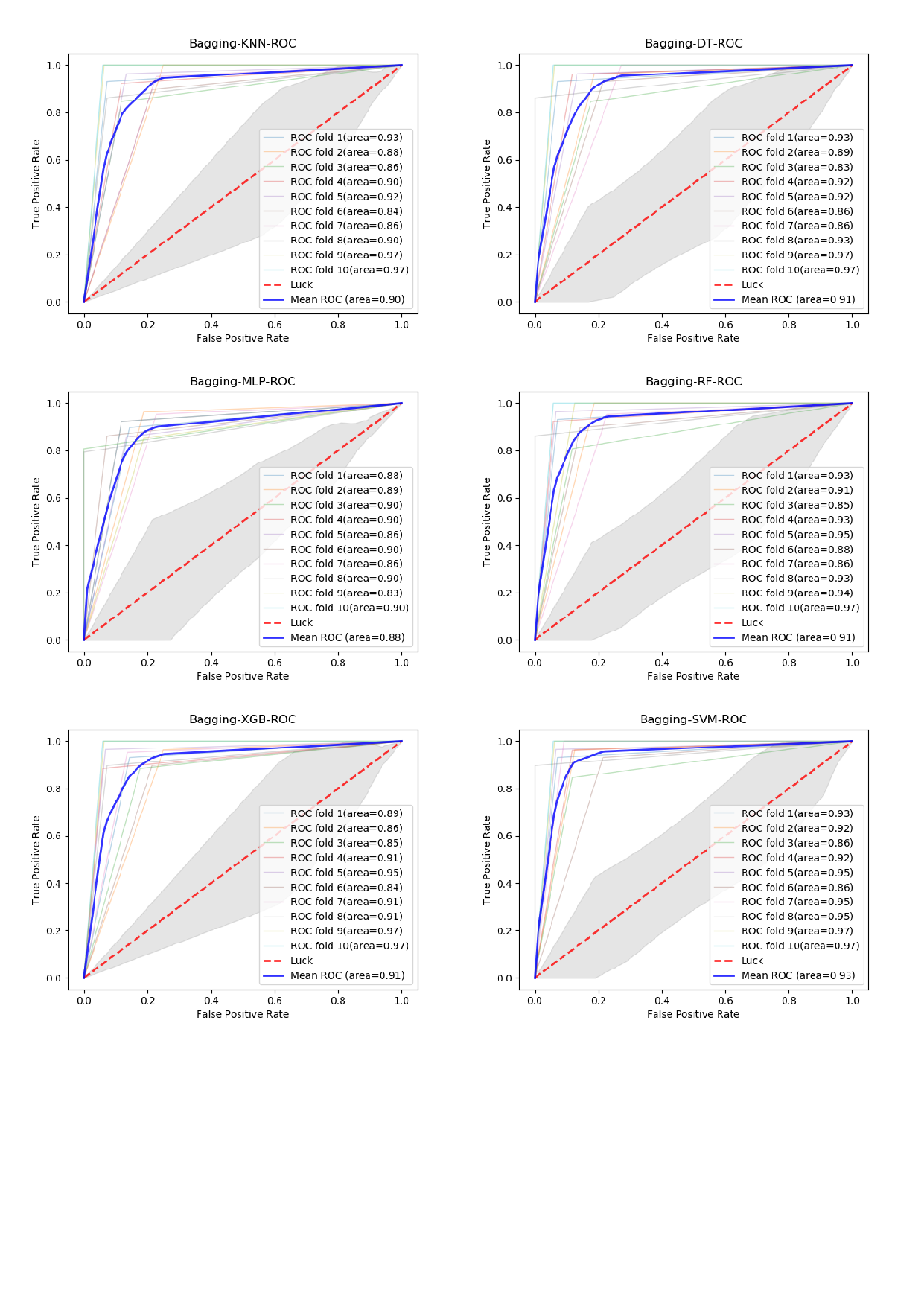

Supplement: Supplementary file 3 [file DataSheet4.zip › Figures/Supplementary Figure S2.tif]
