# Supplementary material for: A method for identifying moonlighting proteins based on linear discriminant analysis and bagging-SVM
Source: Front Genet. 2022 Aug 15;13:963349. doi: 10.3389/fgene.2022.963349 (PMC9420859; doi:10.3389/fgene.2022.963349)
Supplement: Supplementary file 4 [file DataSheet1.pdf]

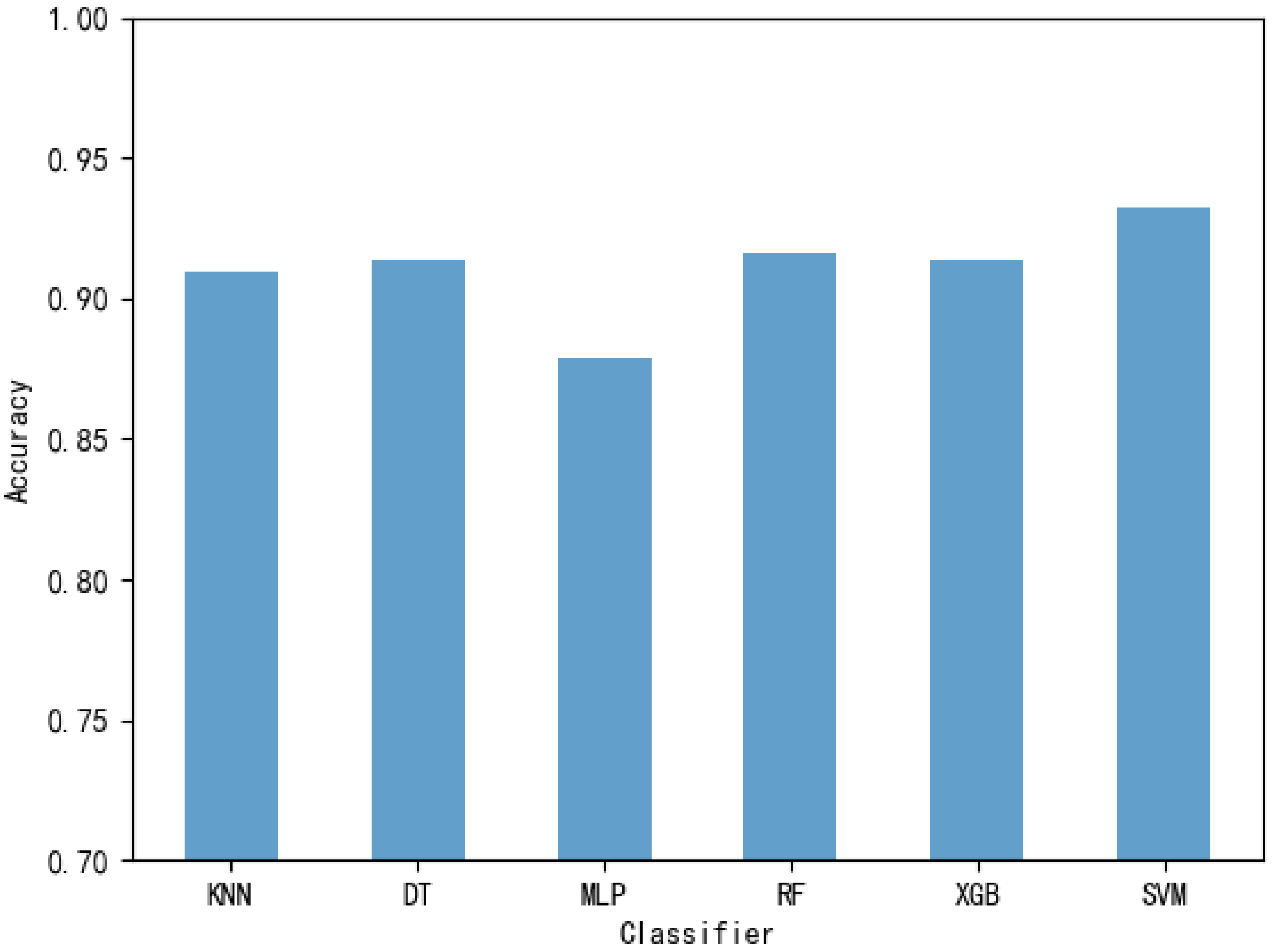

Supplementary Figure S1 The accuracy of Bagging integration for all classifiers on SVMProt-188D.

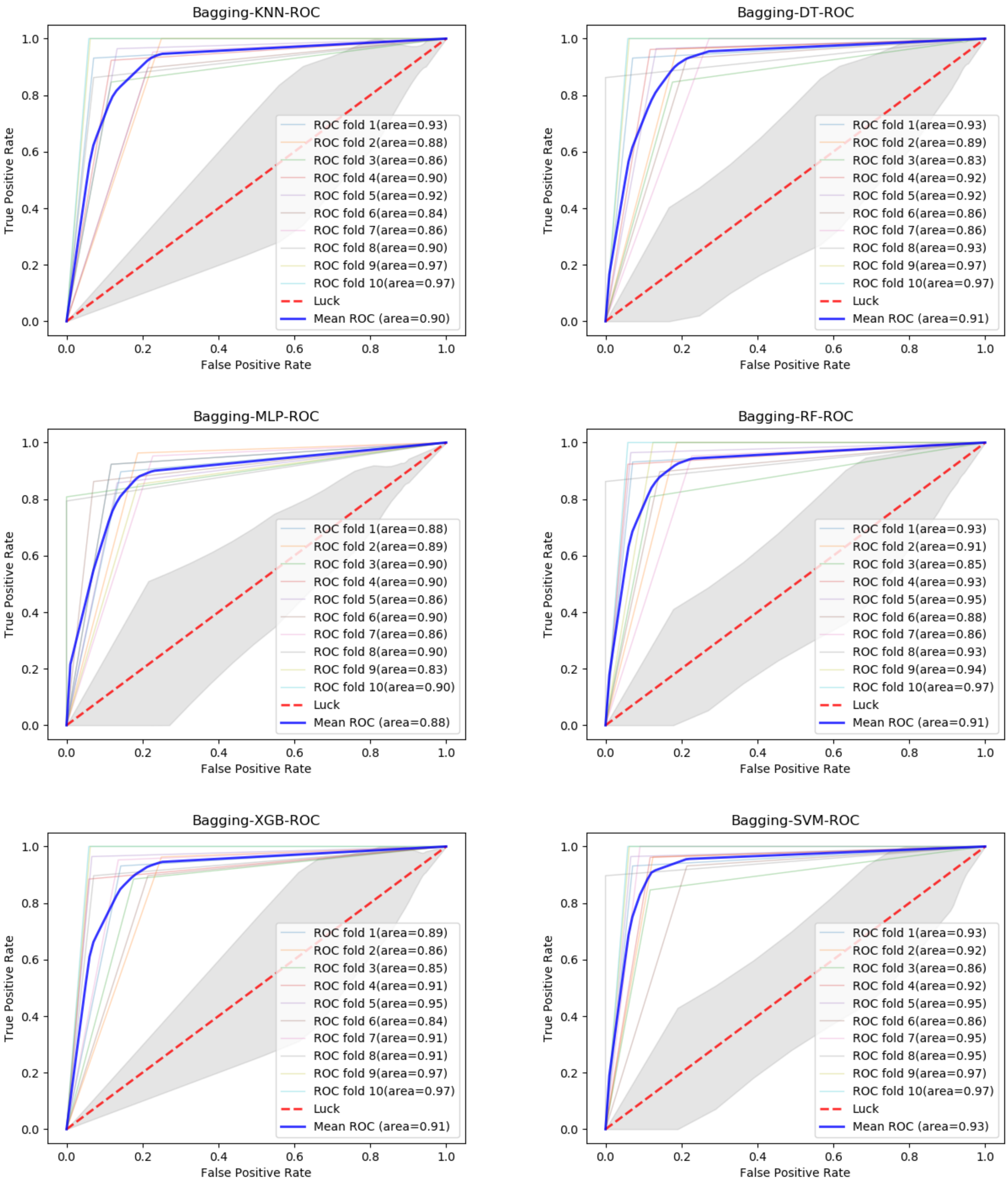

Supplementary Figure S2 The ROC curves of Bagging integration for all classifiers on SVMProt-188D.
